# Developing a medication communication framework across continuums of care using the Circle of Care Modeling approach

**DOI:** 10.1186/1472-6963-13-418

**Published:** 2013-10-17

**Authors:** Nicole A Kitson, Morgan Price, Francis Y Lau, Grey Showler

**Affiliations:** 1 eHealth Observatory, Health Information Science, University of Victoria, STN CSC, PO Box 3050, Victoria, BC V8W 3P5, Canada; 2 Department of Family Practice, University of British Columbia, 3rd Floor David Strangway Building, 5950 University Boulevard, Vancouver, BC V6T 1Z3, Canada

**Keywords:** Medication management, Communication, Action research, Circle of care modeling

## Abstract

**Background:**

Medication errors are a common type of preventable errors in health care causing unnecessary patient harm, hospitalization, and even fatality. Improving communication between providers and between providers and patients is a key aspect of decreasing medication errors and improving patient safety. Medication management requires extensive collaboration and communication across roles and care settings, which can reduce (or contribute to) medication-related errors. Medication management involves key recurrent activities (determine need, prescribe, dispense, administer, and monitor/evaluate) with information communicated within and between each. Despite its importance, there is a lack of conceptual models that explore medication communication specifically across roles and settings. This research seeks to address that gap.

**Methods:**

The Circle of Care Modeling (CCM) approach was used to build a model of medication communication activities across the circle of care. CCM positions the patient in the centre of his or her own healthcare system; providers and other roles are then modeled around the patient as a web of relationships. Recurrent medication communication activities were mapped to the medication management framework. The research occurred in three iterations, to test and revise the model: Iteration 1 consisted of a literature review and internal team discussion, Iteration 2 consisted of interviews, observation, and a discussion group at a Community Health Centre, and Iteration 3 consisted of interviews and a discussion group in the larger community.

**Results:**

Each iteration provided further detail to the Circle of Care medication communication model. Specific medication communication activities were mapped along each communication pathway between roles and to the medication management framework. We could not map all medication communication activities to the medication management framework; we added *Coordinate* as a separate and distinct recurrent activity. We saw many examples of coordination activities, for instance, Medical Office Assistants acting as a liaison between pharmacists and family physicians to clarify prescription details.

**Conclusions:**

Through the use of CCM we were able to unearth tacitly held knowledge to expand our understanding of medication communication. Drawing out the coordination activities could be a missing piece for us to better understand how to streamline and improve multi-step communication processes with a goal of improving patient safety.

## Background

Medication errors are a common type of preventable errors in health care causing unnecessary patient harm, hospitalization, and even fatality [1,2]. The estimated rate of preventable Adverse Drug Events, caused by medication errors, is 1.5 million per year in the United States [2]. There is a link between medication errors and medication communication [2,3]; communication between providers and between providers and patients, within and across care settings, have been identified as sources of medication error [1,2,4-9]. Improving communication is a key aspect of decreasing medication errors and improving patient safety [2,4,10].

**Figure 1:**
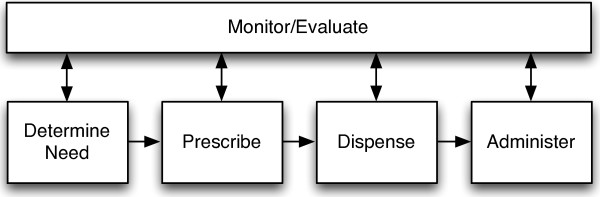
**Medication management framework adapted from **[2,14,15,16,17]**.**

Medication management requires extensive collaboration and communication across roles and care settings [2,10-13], which can reduce (or contribute to) medication-related errors [2,3]. Medication management involves key recurrent activities: determine need; prescribe; dispense; administer; and monitor/evaluate with information communicated within and between each activity [2,14-17]; medication communication is therefore embedded within the framework; whereas, our research seeks to draw it out. The medication management framework is presented in Figure 1.

Despite its importance, communication is often an embedded component (not the focus) of models that explore medication management workflow [14,18], including describing: medicine pathways [14,15]; e-prescribing [16,17]; and patient safety [2,10]. For instance, of the six conceptual models discussed in Liu’s [10] critical review of models to improve patient safety, only one focused specifically on medication management across the continuum of care (the Australian Pharmaceutical Advisory Council’s (APAC) Partnership Model) and one on medication communication specifically (the Medication Communication Model). The APAC Partnership Model [14] outlines nine components for achieving continuity in medication management across the continuum of care. These components are comprised within a medication management cycle and are: decision to prescribe medicine; record of medicine order/prescription; review of medicine order/prescription; issue of medicine; provision of medicine information; distribution and storage; administration of medicine; monitor for response; and transfer of verified information. This model focuses primarily on medication communication between medication management cycles, rather than the communication within the cycle (e.g., between each component). The Medication Communication Model [18] is built from a concept analysis from the literature. The model focuses on three dimensions of face-to-face medication communication: antecedents (sociocultural and environmental factors); attributes (Who is speaking? Who is silent? What is said? What are the prioritized aspects of patient care? What is the body language? What are the actual words used?); and outcomes or consequences (the beneficial or unwanted effects of communication) [18].

While there have been studies on team communication within healthcare departments (for instance, oncology [11,19], Emergency Department [12], Intensive Care Unit [13]), or focusing on specific communication interactions (like between the patient and providers [19,20]), there are fewer studies that explore healthcare communication across the continuum of care or across the patient’s circle of care. The interdisciplinary team communication framework [11] helps fill this gap as it explores healthcare communication structures, processes, and outcomes across continuums of care for palliative care delivery, which can be used to inform the design of Health Information Systems to support communication. In this framework: ‘structure’ refers to the internal (e.g., membership, policies) and external (e.g., contacts, services) structures and their communication channels; ‘process’ refers to care planning, information exchange, teaching, decision making, negotiation, and leadership; and ‘outcomes’ refers to discharge-based (e.g., discharge planning) and patient-based (e.g., satisfaction, goal achievement) outcomes [11].

Our research focuses on medication communication across continuums of care. The medication management framework was used as the foundation for categorizing medication communication activities within the patient’s circle of care. This paper presents a medication communication framework built on the findings gathered from creating a Circle of Care model that explores medication communication.

## Methods

### Approach

The Circle of Care Modeling (CCM) approach was used to build a model of medication communication activities across a patient’s circle of care. CCM positions the patient in the centre of his or her own healthcare system, their circle of care; providers and other roles are then modeled around the patient as a web of relationships. CCM has been used successfully by our team to explore opportunities to improve: continuity of care [21,22]; personal health records (unpublished); and rural patient attachment issues [23]. In this study, CCM was used to identify roles, and medication communication pathways and activities within the patient’s circle of care, to highlight gaps and challenges, and reason about possible improvements. Our analysis of these activities was both deductive, as we mapped to existing medication management activities, and inductive, as we drew out specific communication activities not reflected in the medication management framework.

The research occurred in three iterations: Iteration 1 developed an initial Circle of Care medication communication model based on a review of the literature; Iteration 2 gathered field data at a Community Health Centre (CHC) with an integrated pharmacy; and Iteration 3 gathered data within a broader urban community. The Circle of Care medication communication model was revised in each iteration.

### Iteration 1: Theoretical

#### Participants and recruitment

None.

#### Data collection

The research team conducted a *literature scoping review* in December 2010 to explore medication communication pathways and activities. Medline, CINAHL, and Google Scholar were searched from 1998–2010 using the following search terms: medication communication, medication management, medication error, patient safety, and/or ambulatory care. The articles were included if they described medication communication pathways and/or activities between care providers or patients. Forty-five articles were retrieved. An internal *discussion* with five members of the research team (one physician, two registered nurses, and two health informaticians) explored additional medication communication pathways and activities. In this context, a pathway is the direction of communication between individual roles (the part someone plays in the patient’s circle of care); communication from nurse to doctor and doctor to nurse represents two pathways. Activities refer to medication communication activities using the activities reflected in the medication management framework as an initial basis for identification and classification (e.g., prescribe, dispense, administer).

#### Data analysis

We drew medication communication activities and pathways from the *literature* to create an initial Circle of Care medication communication model. Additional pathways were added after a team *discussion*. The team *discussion* validated the draft Circle of Care medication communication model and informed the development of a medication communication framework. This information was used as a basis for creating patient personas (simulated patient cases) to discuss with participants in subsequent research iterations.

### Iteration 2: Integrated community health centre

#### Participants and recruitment

Recruitment occurred at a single inner city integrated Community Health Centre (CHC) and included patients and care providers. Patients were recruited by a poster in the waiting room; interested patients identified themselves to the research nurse who asked further questions for eligibility: a patient at the CHC for at least one year, currently taking at least three prescribed medications dispensed by the CHC pharmacy (compliance and other factors were not part of the inclusion criteria). Patient participants were provided a gift card for participation. Provider participants were recruited by the CHC manager and included family physicians, registered nurses, pharmacy employees, and Medical Office Assistants (MOAs). MOAs are administrative office assistants in a medical or healthcare environment. Provider participants identified themselves to the researchers directly and chose to participate in an interview, observation, and/or discussion.

#### Data collection

We used mixed methods, including: observation, patient interviews, provider interviews, and provider discussion. *Observation* occurred with consenting patients and care providers within the CHC. Observation of patient/clinician interactions focused on medication communication only and took approximately ten minutes per patient; the researcher left the room when asked. Clinician observation occurred in three-hour time blocks. No patient identifiable information was documented. Detailed notes were taken on medication communication and included roles (e.g., doctor, patient), medication communication activity (e.g., request new prescription), and communication pathway (e.g., patient → physician). *Patient interviews* consisted of semi-structured questions to explore the roles and content of medication communication. *Provider interviews* were structured around two patient personas that were developed after Iteration 1 to highlight common medication communication scenarios and challenges. Interviews were audio-recorded and detailed notes were taken. No personal information was recorded. The one-hour *provider discussion* was characterized by active discussion with multiple roles and perspectives; the group reviewed the model to confirm accuracy, and discussed potential local (internal) improvements to medication communication. The *discusison* was audio-recorded and detailed notes were taken.

#### Data analysis

*Observation* notes were reviewed to identify specific communication pathways and activities between roles. *Interviews* were transcribed and thematically coded, along with the detailed notes. Several elements were extracted including roles, pathways, recurrent medication communication activities, and challenges encountered in medication communication. The observations and interviews collectively were used to inform the Circle of Care medication communication model. Recurrent medication communication activities were mapped to the medication management framework. The *discussion* audio-recordings and detailed notes were also thematically coded as above. Multiple perspectives (patients, providers) served to triangulate the findings. The findings from iteration 2 were used to inform iteration 3.

### Iteration 3: Community

#### Participants and recruitment

A standard recruitment letter was sent to physician offices and community-pharmacies listed in the 2011 Physician and Surgeons Directory for Victoria, Saanich and the Gulf Islands (excluding the Gulf Islands). Research assistants followed up with potential participants in person and via fax. Community Nurses were recruited through Home and Community Care (HCC) at Vancouver Island Health Authority (VIHA).

#### Data collection

In this iteration, one-hour *interview*s were conducted with providers only. Interviews were structured around two patient personas (simulated patient cases) to stimulate discussion about medication communication activities across circles of care. One patient persona was reused from Iteration 2; whereas, a second one was created to better reflect a typical patient seen in the community (rather than the inner city CHC). Interviews were audio-recorded and detailed notes were taken. The two-hour *discussion* involved bringing together providers from both iterations two and three to discuss the Circle of Care medication communication model and medication communication framework developed during the project, and to identify opportunities to improve medication communication. There were no observation sessions in iteration 3. The discussion was audio-recorded and detailed notes were taken.

#### Data analysis

As in Iteration 2, the rich set of data from *interviews* and the *discussion* were translated visually into a refined and expanded rich Circle of Care medication communication model. Recurrent medication communication activities were mapped to the medication management framework.

### Synthesis of findings

Data collection activities and analyses were iterative, and used to inform the medication communication framework and the Circle of Care medication communication model. Each of the iterations built upon the findings of the preceding iteration(s), with Iteration 2 informed by the findings of Iteration 1, and Iteration 3 informed by the combined findings of Iterations 1 and 2. These iterative findings collectively contributed to what we know about medication communication within and between roles in the patient’s circle of care. The combined findings from Iterations 1, 2, and 3 are presented in the Results section below.

Ethics approval was granted by the University of Victoria (UVic), protocol number: 11–093 and UVic/Vancouver Island Health Authority, protocol number: J2011-54.

Patient personas and the interview question tool are freely available at http://ehealth.uvic.ca/initiatives/projects/pharmacy.php.

## Results

### Participants

The research occurred from April 2011 to May 2012. A total of 39 patients and community care providers (representing 8 roles) participated in the research; some participants were involved in multiple activities (e.g., observation, interview, and discussion). The breakdown of participant roles by data collection method is presented in Table 1.

**Table 1:** Breakdown of participant roles for Iterations 2 or 3

	**Iteration 2**			**Iteration 3**	
**Role**	**Observation**	**Interview**	**Discussion**	**Interview**	**Discussion**
Family Physician	2	3	4	5	2
Home and Community Care Nurse	0	0	0	3	1
Medical Office Assistant	3	2	5	1	2
Community Health Centre Nurse	2	2	3	0	1
Patient	3	6	0	0	0
Pharmacist	1	1	1	4	2
Pharmacy Technician	2	1	2	1	1
Specialist Physician	0	0	0	3	1
Administrator	0	0	1	0	0
**Total**	**13**	**15**	**16**	**17**	**10**

### The medication communication framework

**Figure 2:**
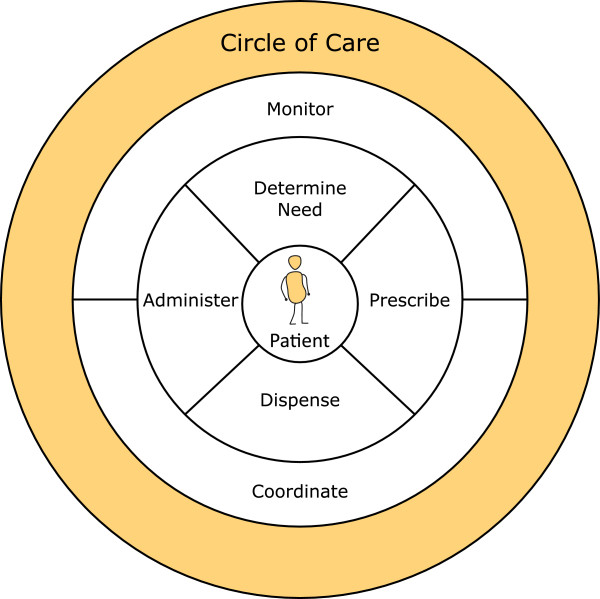
Medication communication framework.

While the five medication management categories (determine need, prescribe, dispense, administer, monitor/evaluate) had alignment with communication activities, not all communication activities could be mapped directly to the medication management framework (Figure 1). Our research highlighted *Coordinate* as a separate and distinct recurrent activity. We observed many instances of specific roles (e.g., MOA, HCC liaison, HCC nurse, pharmacy technician) coordinating medication information between providers, and between providers and patients. The medication communication framework is presented in Figure 2.

Medication communication activities are defined below:

● **Determine need** involves communication around activities to determine the need for medication, e.g., a doctor and patient discussing the patient’s complaint;

● **Prescribe** focuses on communication around prescribing activities, e.g., a patient and doctor discussing the details of a new prescription;

● **Dispense** focuses on communication around dispensing activities, e.g., a pharmacist and physician resolving an alert for a duplicate medication;

● **Administer** involves communication around medication administration activities, e.g., a pharmacist and patient discussing medication administration instructions;

● **Monitor/Evaluate** focuses on communication around medication monitoring and evaluation, e.g., a patient and Home and Community Care nurse discussing medication compliance. The monitor/evaluate activity was often used to inform the other medication communication activities for medication decision-making; and

● **Coordinate communication** focuses on the coordination of medication information between roles, e.g., an MOA transmitting a request for information between a pharmacist and a family physician.

The number of roles, number of pathways, and list of sub-activities for each of these medication communication activities is in Table 2.

**Table 2:** Roles, pathways, and sub-activities for medication communication activities

**Communication activity**	**Roles**	**Pathways**	**Sub-activities**
Determine need	20	54	•Discuss complaint
			•Discuss social context
			•Discuss non-medicinal and medicinal options
			•Discuss plans and goals
			•Educate
Prescribe	16	46	•Confirm patient identity
			•Provide medication information
			•Request, prepare, confirm, alert prescription/renewal
			•Request, confirm emergency supply of medications
			•Review coverage
			•Request, discuss restrictions
			•Review prescribing alerts
Dispense	14	36	•Confirm patient identity
			•Provide medication information
			•Confirm medication availability
			•Request, prepare, confirm, alert dispensing
			•Review coverage
			•Request, discuss restrictions
			•Review, notify dispensing alerts
Administer	12	28	•Provide medication administration instructions
			•Schedule medication administration
			•Request, prepare, modify, clarify Delegation of Task (for Home and Community Care)
Monitor/Evaluate	47	200	•Confirm, request, review current medication details
			•Confirm, request, review past medication details
			•Discuss medication compliance
			•Request, provide, confirm allergy information
			•Discuss experience of side effects
			•Review medication efficacy
			•Request, confirm appointment
			•Request, order, review tests
			•Review self-monitoring
			•Confirm, request, review care transitions
Coordinate	15	36	•Request, confirm appointments and referrals
			•Request, transmit patient information
			•Relay messages between patients and care providers
			•Request, confirm, alert coverage

### Coordinate

We identified 36 communication pathways between 15 roles for the Coordinate medication communication activity. However, given the structure of our research enquiry (for instance, extended observation of MOA medication communication activities) this paper focuses more specifically on our coordinate communication activity findings in the context of the MOA role. We observed MOAs as information conduits, transmitting medication information in support of medication management in the patient’s circle of care.

**Figure 3:**
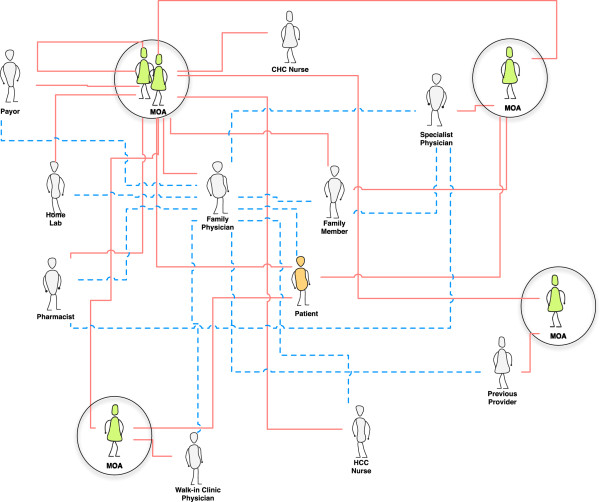
**A view of the Circle of Care Model that highlights only the coordinate communication activity.** Solid lines represent direct pathways of communication; whereas dotted lines reflect indirect connections. Abbrev: Medical Office Assistant (MOA); Home and Community Care (HCC).

Coordinating activities include: relaying messages between care providers and relaying messages between patients and care providers; requesting or transmitting patient information for current or historical medication records; requesting and confirming appointments and referrals; and requesting, confirming, or alerting roles of the status of medication coverage. While the content of what these roles communicate falls within other medication communication activities (e.g., prescribing, dispensing, monitoring), their responsibility is to facilitate the coordination of this information between roles (e.g., between a family physician and a pharmacist). Figure 3 highlights the MOA pathways for the coordinate communication activity within the Circle of Care medication communication model.

Figure 3 highlights the coordinate communication activity in the Circle of Care Model. There are 36 direct and 22 indirect pathways between 15 roles: the patient; physicians (family physician, specialist physician, walk-in clinic physician, previous provider) and their Medical Office Assistants; nurses (HCC, CHC); pharmacist; family member; home lab; and the Payor. Additional roles and pathways could exist. Direct pathways represent those roles MOAs have direct communication with to coordinate medication information. Indirect pathways indicate those connections that can be made between roles as a result of the coordinating activity. For instance, a walk-in clinic physician can communicate with a patient’s family physician that a new prescription was provided; this information is transmitted via the walk-in clinic physician’s MOA and the family physician’s MOA.

Interestingly, we observed fewer coordinate communication pathways in the integrated clinic versus the non-integrated clinics. In a non-integrated clinic, the pharmacy is off-site and coordination is regularly required to link the family physician and pharmacist together to confirm, for instance, current medications as reflected in the electronic provincial medication repository or to discuss a dispensing alert (e.g., for a contraindicated medication). In the integrated clinic with a pharmacy on-site, however, conversations between the pharmacist and family physician generally occur directly in the pharmacy or clinic hallway.

### Circle of Care medication communication model

**Figure 4:**
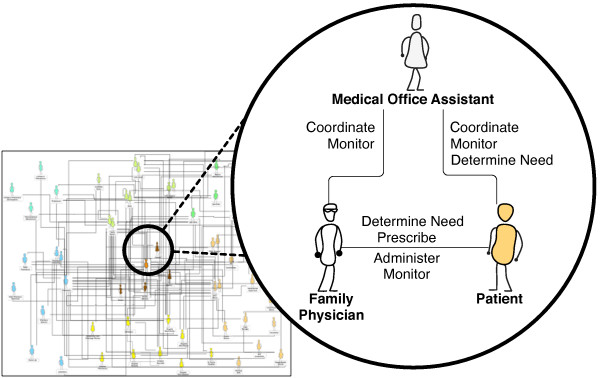
Circle of Care medication communication model.

The medication communication activities were mapped along pathways between roles. This is illustrated by the Circle of Care Medication Communication Model (Figure 4). The Circle of Care medication communication model grew from an initial 50 communication pathways between 11 roles from the literature to an intricate web of 252 communication pathways between 50 roles (doctor to nurse, nurse to doctor counts as 2 pathways). In order to capture communication pathways between providers and sites, we made a distinction between, for instance, the patient’s primary family physician, and a family physician at a walk-in clinic. Similarly, we captured the pharmacy staff at the patient’s primary pharmacy, as well as a secondary pharmacy.

In terms of roles and medication communication pathways, 11 of the 50 roles communicated with more than 5 other roles, they are: patient (34 other roles), family physician (28), pharmacist (22), HCC nurse (15), MOA (13), specialist physician (12), CHC nurse (12), family member (8), hospital ward doctor (8), hospital ward nurse (7), pharmacy technician (7), and Emergency Room (ER) doctor (6). These roles largely had pathways that interconnected with each other. Most roles (39 of 50) shared communication pathways with 5 or fewer roles. Examples of these roles are HCC home workers, case workers, support groups, and laboratory technicians. These pathways primarily connected with the patient, family physician, pharmacist, and MOA.

The combined list of roles and communication pathways for all medication communication activities, along with roles and communication pathways for specific medication communication activities, are provided as online Additional file 1 and Additional file 2.

## Discussion

### The medication communication framework

The Circle of Care medication communication model places the patient at the centre of his or her own circle of care; family members, providers, and other roles are then modeled around the patient as they collectively engage in medication communication activities. The deductive and inductive data analysis helped us to recognize, draw out, and classify recurrent medication communication activities, and related roles and pathways within the circle of care. In addition to the activities that could be mapped to the medication management framework (determine need, prescribe, dispense, administer, monitor/evaluate), we identified Coordination of medication information as a key recurrent activity that may have been previously overlooked.

There are similarities and differences between our medication communication framework, Manias’ Medication Communication Model [18], and Kuziemsky’s interdisciplinary team communication framework [11]. In terms of the Medication Communication Model [18], our research explored some of the sociocultural and environmental factors (antecedents) influencing medication communication and outcomes of current (and potentially future) practice; in particular, we explored gaps in medication communication, underlying challenges, and potential opportunities to improve (these were out of scope for this paper). We also explored the attributes of ‘who is speaking to whom about what’ when it comes to medication communication; however, we did not specifically explore body language, the aspect of silence, or the actual words used. Another difference is that our framework incorporates both asynchronous and synchronous communication pathways; whereas, the Medication Communication Model focuses on face-to-face communication. In terms of Kuziemsky’s interdisciplinary team communication framework [11], our framework incorporates aspects of internal and external structure (members of the care team, procedures, contacts) and communication channels. There is alignment between the interdisciplinary team communication processes, and the medication communication activities we identified (e.g., care planning and teaching are part of communicating about Determine Need). Unlike Kuziemsky, however, our enquiry did not explore discharge planning specifically. Similarly, our outcomes were focused on improving patient safety through improved medication communication; whereas, Kuziemsky’s framework looked at broader patient- and discharge-based outcomes. The medication communication framework we created can draw from both the Medication Communication Model and interdisciplinary team communication framework to add further depth and breadth to our understanding of medication communication within teams and across the continuum of care.

### Implications for practice and research

This research can help improve medication communication activities within and between provider practices to improve quality of patient care. Practitioners can draw from the medication communication resources we developed as a guide to: identify specific coordination activities (and the roles responsible for them) within their organizations as they relate to medication communication; and to explore potential consequences (both good and bad) of changing existing medication communication practices. The guides can also be used broadly as a basis for identifying the opportunities to improve medication communication activities across the patient’s circle of care.

Researchers can help fill a gap in knowledge by designing future studies to explore medication communication across the continuum of care; these studies can expand, validate, and revise the resources we provide in this paper, including exploring the differences in medication communication pathways between integrated and non-integrated clinics. We have only scratched the surface to explore the coordinating activity for medication communication; additional studies could provide further insight. The use of observation was an essential component of being able to unearth this tacitly held knowledge [24]. Organizational communication practices are interactive and socially embedded [25]; indirect pathways, such as those reflected in the coordinate communication activity (and other tacit day-to-day activities), may be taken for granted, but could be equally important in terms of their potential for error or improvement.

### Contribution to new knowledge

This patient-centric research contributes to the knowledge of what is known about medication communication across the continuum of care. We built a medication communication framework, a Circle of Care medication communication model, and a taxonomy of medication communication; future studies can expand, validate, and revise these resources. Further, this research fills a gap in knowledge by drawing attention to the ‘coordinate communication’ activity. This activity has the potential to be taken for granted as it can represent an embedded process that can be institutionalized over time.

### Limitations

This study was limited to community providers and patients within one city. Further, observation of communication activities occurred within the Community Health Centre only and not within the larger community itself. We used a limited set of simulated patient cases (three). Additional cases or characteristics could reveal other communication pathways and activities not yet represented in the model, for instance, further exploring transitions between the community, acute care, and long-term care facilities. This study was exploratory and qualitative; we did not attempt to quantify the frequency or impact of the various communication activities.

## Conclusions

Through the use of an exploratory approach, Circle of Care Modeling, we were able to unearth tacitly held knowledge to expand our understanding of medication communication across the continuum of care. This knowledge can be used to improve the quality of patient care through the identification and improvement of medication communication gaps and the design of health information systems (HIS). Drawing out the coordinate communication activities in the medication communication framework could be a missing piece for us to better understand how to streamline multi-step processes. Better understanding of this activity could also help us anticipate some of the unintended consequences of improvement processes, for instance, if tacit activities are not taken into account.

## Consent

Written informed consent was obtained from the patient for the publication of this report and any accompanying images.

## Competing interests

The authors declare that they have no competing interests.

## Authors’ contributions

NK carried out the fieldwork and drafted the manuscript. MP and FL conceived of the study. MP designed and coordinated the study, and drafted the manuscript. GS engaged in the initial literature review and participated in persona design. FL provided detailed feedback to the manuscript. All authors read and approved the final manuscript.

## Pre-publication history

The pre-publication history for this paper can be accessed here:

http://www.biomedcentral.com/1472-6963/13/418/prepub

## Supplementary Material

Additional file 1 Supplementary materials: Taxonomy of medication and communication activities. Click here for file

Additional file 2 Supplementary materials: Medication communication activities - roles and pathways. Click here for file
