# Supplementary material for: Developing a medication communication framework across continuums of care using the Circle of Care Modeling approach
Source: BMC Health Serv Res. 2013 Oct 17;13:418. doi: 10.1186/1472-6963-13-418 (PMC3853098; doi:10.1186/1472-6963-13-418)
Supplement: Additional file 1 — Supplementary materials: Taxonomy of medication and communication activities. [file 1472-6963-13-418-S1.pdf]

Supplementary Materials
Taxonomy of Medication Communication Activities

Nicole A. Kitson, PhD, Morgan Price, MD, PhD, Francis Lau, Grey Showler, RN, BSN
Developing a medication communication framework across continuums of care using the Circle of Care Modeling approach, BMC Health Services Research, 2013.

Communicate – DETERMINE NEED

Discuss complaint	• Describe symptoms<br>• Discuss condition<br>• Review history<br>• Discuss personal information<br>• Provide advocacy<br>• Give history
Discuss social context	• Finances<br>• Housing<br>• Social history
Discuss non-medicinal options	
Discuss medicinal options	
Discuss plans and goals	• Discuss health goals<br>• Discuss patient needs<br>• Set expectations<br>• Build a problem list<br>• Create, discuss, review, revise plans<br>o Shared care<br>o Patient care<br>o Self-monitoring plan<br>o Discharge plan<br>o Medication plan<br>• Discuss attachment to Family Physician or clinic
Educate	• Share medication experience<br>• Encourage best practice<br>o See same doctor or go to same clinic<br>o Fill at one pharmacy<br>o Return unused medication<br>o Do not share medications with friends<br>• Interpret scientific report or medication information from Internet

Communicate – PRESCRIBE

Confirm patient identity	
Provide medication information	• Dose, frequency, route, timing<br>• Name of medication<br>• Benefits and risks<br>• Side effects
Request, prepare, confirm, alert prescription	• New prescription<br>• Modified prescription<br>○ Change medication<br>○ Discontinue medication<br>○ Hold medication<br>○ Stop, cancel medication<br>○ Adjust dosage<br>• Renewal prescription
Request, confirm emergency supply of medications	
Review coverage	• Discuss medication coverage<br>• Request, confirm, alert special authority
Request, discuss restrictions	• Frequency of pickups<br>• No early refills<br>• No refills for lost prescriptions<br>• Limit to one pharmacy<br>• Medication type and quantity
Review prescribing alerts	• Drug-to-drug interactions<br>• Allergies<br>• Duplication therapy<br>• Dosing errors<br>• Contraindications

Communicate – DISPENSE

Confirm patient identity	
Provide medication information	• Dose, frequency, route, timing<br>• Name of medication<br>• Benefits and risks<br>• Side effects
Confirm medication availability	
Request, prepare, confirm, alert dispensing	• New prescription<br>• Modified prescription<br>o Change medication<br>o Discontinue medication<br>o Hold medication<br>o Stop, cancel medication<br>o Adjust dosage<br>• Renewal prescription<br>o Alert refills remaining<br>• Confirm packaging<br>• Confirm, modify dispensing schedule<br>• Request, confirm status of dispensing<br>• Request, provide harm reduction supplies<br>• Provide patient medication delivery info<br>• Redirect patient to care provider
Review coverage	• Discuss medication coverage<br>• Request, confirm, alert special authority<br>• Request, confirm insurance information<br>• Schedule medication payment<br>• Discuss cost of medication<br>• Discuss dispensing fees<br>• Discuss ability to pay<br>• Alert medication not covered
Request, discuss restrictions	• Frequency of pickups<br>• No early refills<br>• No refills for lost prescriptions<br>• Limit to one pharmacy<br>• Medication type and quantity
Review, notify dispensing alerts	• Drug-to-drug interactions<br>• Allergies<br>• Duplication therapy<br>• Dosing errors<br>• Contraindications

Communicate – ADMINISTER

Provide medication administration instructions	• Initial instructions<br>○ Timing<br>• Revised instructions<br>○ Change timing of medication<br>○ Titration<br>• Confirm/clarify instructions
Schedule medication administration	
Request, prepare, modify, clarify Delegation of Task	

Communicate – MONITOR/EVALUATE

Confirm, request, review current medication details	Name, dose, route, dispensing schedule, prescribers, dispensers, frequency<br>• Community<br>• Hospital<br>• Samples<br>• Over-the-counter
Confirm, request, review past medication details	
Discuss medication compliance	• Current compliance<br>• Past compliance
Request, provide, confirm allergy information	
Discuss experience of side effects	
Review medication efficacy	
Request, confirm appointment	• Doctor<br>• Hospital<br>• Specialist
Request, order, review tests	• Request lab tests, bloodwork order<br>• Order lab tests, bloodwork, UDS<br>• Discuss, communicate lab results, values<br>• Confirm reason for lab order<br>• Discuss bloodwork schedule
Review self-monitoring	• Instructions<br>• Report results<br>• Request, confirm, deny share medications<br>• Review symptoms (e.g., of low blood sugar)
Confirm, request, review care transitions	• Request, provide report<br>• Notify patient admitted to/discharged from hospital<br>• Confirm provider details<br>• Confirm dispenser details<br>• Request follow-up with patient

COORDINATE Communication

Request, confirm appointments/referrals	• Prescription<br>• Lab test, bloodwork<br>• Specialist<br>• Doctor
Request, transmit patient information	• Current medical records<br>• Historical medical records<br>• Release forms
Relay messages between patients and care providers	
Request, confirm, alert coverage	• Includes Special Authority
