# Supplementary material for: Developing a medication communication framework across continuums of care using the Circle of Care Modeling approach
Source: BMC Health Serv Res. 2013 Oct 17;13:418. doi: 10.1186/1472-6963-13-418 (PMC3853098; doi:10.1186/1472-6963-13-418)
Supplement: Additional file 2 — Supplementary materials: Medication communication activities - roles and pathways. [file 1472-6963-13-418-S2.pdf]

Supplementary Materials
Medication Communication Activities – Roles and Pathways

Nicole A. Kitson, PhD, Morgan Price, MD, PhD, Francis Lau, Grey Showler, RN, BSN
Developing a medication communication framework across continuums of care using the Circle of Care Modeling approach, BMC Health Services Research, 2013.

Medication Communication Activity Tables:

- Combined activities
- Determine Need
- Prescribe
- Dispense
- Administer
- Monitor/evaluate
- Coordinate

**Medication Communication Activity: Roles and Pathways. Represents 252 pathways between 50 roles.** Light blue boxes represent one connection (e.g., Patient --> Medical Office Assistant); whereas, dark blue boxes represent two connections within roles (e.g., communication between two (or more) Medical Office Assistants within the same clinic).	Patient	Family physician	Specialist physician	Walk-in clinic doctor	Previous provider	Doctor on call	Pharmacist	Pharmacy technician	Pharmacy delivery	Pharmacist (other pharmacy)	Pharmacy technician (other pharmacy)	Medical Office Assistant	Specialist Medical Office Assistant	Walk-in clinic Medical Office Assistant	Previous Provider Medical Office Assistant	Family member	Friends	Partner	Home and Community Care Nurse	Community Health Centre Nurse	Outreach nurse	Corrections nurse	Emergency Room doctor/Physician on duty	Hospital ward doctor	Hospital ward nurse	Hospital specialist	Hospital Home and Community Care Liaison	Community care discharge planner	Hospital records	Hospital pharmacist	Laboratory	Home laboratory services	Acute residential care	Longterm care	Home worker	Home worker supervisor	Community Health Centre Support Group	Support Group	Social worker	Case manager	Assertive Community Treatment (ACT) Caseworker	Counselor	Mental Health worker	Housing staff	Payor	St. Paul's Hospital	College of Physicians and Surgeons	College of Pharmacists	Pharmaceutical Manufacturers	Researcher	
**Patient**																																																			34
**Family physician**																																																			28
**Specialist physician**																																																			12
**Walk-in clinic doctor**																																																			4
**Previous provider**																																																			2
**Doctor on call**																																																			1
**Pharmacist**																																																			22
**Pharmacy technician**																																																			7
**Pharmacy delivery**																																																			2
**Pharmacist (other pharmacy)**																																																			4
**Pharmacy technician (other pharmacy)**																																																			2
**Medical Office Assistant**																																																			13
**Specialist Medical Office Assistant**																																																			4
**Walk-in clinic Medical Office Assistant**																																																			3
**Previous provider Medical Office Assistant**																																																			2
**Family member**																																																			8
**Friends**																																																			1
**Partner**																																																			1
**Home and Community Care Nurse**																																																			15
**Community Health Centre Nurse**																																																			12
**Outreach nurse**																																																			2
**Corrections nurse**																																																			1
**Emergency Room doctor/Physician on duty**																																																			6
**Hospital ward doctor**																																																			8
**Hospital ward nurse**																																																			7
**Hospital specialist**																																																			5
**Hospital Home and Community Care Liaison**																																																			1
**Community care discharge planner**																																																			1
**Hospital records**																																																			4
**Hospital pharmacist**																																																			1
**Laboratory**																																																			3
**Home laboratory services**																																																			2
**Acute residential care**																																																			1
**Longterm care**																																																			1
**Home worker**																																																			3
**Home worker supervisor**																																																			2
**Community Health Centre Support Group**																																																			3
**Support Group**																																																			1
**Social worker**																																																			2
**Case manager**																																																			2
**Assertive Community Treatment (ACT) Caseworker**																																																			2
**Counselor**																																																			1
**Mental health worker**																																																			3
**Housing staff**																																																			1
**Payor**																																																				5
**St. Paul's Hospital**																																																			2
**College of Physicians and Surgeons**																																																			1
**College of Pharmacists**																																																			1
**Pharmaceutical Manufacturers**																																																			2
**Researcher**																																																			1
**Total connections**																																																			252

**Medication Communication Activity - Communicate DETERMINE NEED: Roles and Pathways. Represents 54 pathways between 20 roles.** Light blue boxes represent one connection (e.g., Patient --> Medical Office Assistant); whereas, dark blue boxes represent two connections within roles (e.g., communication between two (or more) Medical Office Assistants within the same clinic).	Patient	Family physician	Specialist physican	Walk-in clinic doctor	Doctor on call	Pharmacist	Pharmacist (other pharmacy)	Medical Office Assistant	Home and Community Care Nurse	Community Health Centre Nurse	Outreach nurse	Emergency Room doctor/]Physician on duty	Hospital ward doctor	Hospital ward nurse	Hospital specialist	Community care discharge planner	Community Health Centre Support Group	Support Group	Case manager	Mental Health worker	
**Patient**																					**14**
**Family physician**																					**10**
**Specialist physician**																					**4**
**Walk-in clinic doctor**																					**1**
**Doctor on call**																					**1**
**Pharmacist**																					**1**
**Pharmacist (other pharmacy)**																					**1**
**Medical Office Assistant**																					**1**
**Home and Community Care Nurse**																					**3**
**Community Health Centre Nurse**																					**5**
**Outreach nurse**																					**1**
**Emergency Room doctor/Physician on duty**																					**1**
**Hospital ward doctor**																					**1**
**Hospital ward nurse**																					**1**
**Hospital specialist**																					**1**
**Community care discharge planner**																					**1**
**Community Health Centre Support Group**																					**2**
**Support Group**																					**1**
**Case manager**																					**2**
**Mental health worker**																					**2**
**Total connections**																					**54**

**Medication Communication Activity - Communicate PRESCRIBE: Roles and Pathways. Represents 46 pathways between 16 roles.** Light blue boxes represent one connection (e.g., Patient --> Medical Office Assistant); whereas, dark blue boxes represent two connections within roles (e.g., communication between two (or more) Medical Office Assistants within the same clinic).	Patient	Family Physician	Specialist physician	Walk-in doctor	Doctor on call	Pharmacist	Pharmacy technician	Home and Community Care Nurse	Community Health Centre Nurse	Emergency Room doctor/physician on duty	Hospital ward doctor	Hospital ward nurse	Hospital specialist	Community Health Centre Support Group	Payor	Pharmaceutical Manufacturers	
**Patient**																	**9**
**Family physician**																	**7**
**Specialist physician**																	**3**
**Walk-in clinic doctor**																	**2**
**Doctor on call**																	**1**
**Pharmacist**																	**6**
**Pharmacy technician**																	**1**
**Home and Community Care Nurse**																	**1**
**Community Health Centre Nurse**																	**3**
**Emergency Room doctor/physician on duty**																	**2**
**Hospital ward doctor**																	**2**
**Hospital ward nurse**																	**2**
**Community Health Centre Support Group**																	**2**
**Hospital specialist**																	**1**
**Payor**																	**3**
**Pharmaceutical Manufacturers**																	**1**
																	**46**

**Medication Communication Activity - Communicate DISPENSE: Roles and Pathways. Represents 36 pathways between 14 roles.** Light blue boxes represent one connection (e.g., Patient --> Medical Office Assistant); whereas, dark blue boxes represent two connections within roles (e.g., communication between two (or more) Medical Office Assistants within the same clinic).	**Patient**	**Family physician**	**Specialist physician**	**Walk-in clinic doctor**	**Pharmacist**	**Pharmacy technician**	**Pharmacy delivery**	**Pharmacist (other pharmacy)**	**Pharmacy technician (other pharmacy)**	**Home and Community Care Nurse**	**Emergency Room doctor/Physician on duty**	**Hospital ward doctor**	**Hospital ward nurse**	**Pharmacare**	
**Patient**															**5**
**Family physician**															**3**
**Specialist physician**															**1**
**Walk-in clinic doctor**															**1**
**Pharmacist**															**11**
**Pharmacy technician**															**5**
**Pharmacy delivery**															**2**
**Pharmacist (other pharmacy)**															**1**
**Pharmacy technician (other pharmacy)**															**1**
**Home and Community Care Nurse**															**1**
**Emergency Room doctor/Physician on duty**															**1**
**Hospital ward doctor**															**1**
**Hospital ward nurse**															**1**
**Pharmacare**															**2**
	5	3	1	1	11	5	2	1	1	1	1	1	1	2	**36**

**Medication Communication Activity - Communicate ADMINISTER: Roles and Pathways. Represents 28 pathways between 12 roles.** Light blue boxes represent one connection (e.g., Patient --> Medical Office Assistant); whereas, dark blue boxes represent two connections within roles (e.g., communication between two (or more) Medical Office Assistants within the same clinic).	**Patient**	**Family physician**	**Specialist physician**	**Pharmacist**	**Pharmacist (other pharmacy)**	**Home and Community Care Nurse**	**Community Health Centre Nurse**	**Hospital ward doctor**	**Hospital ward nurse**	**Home worker**	**Home worker supervisor**	**Social worker**	
**Patient**													**9**
**Family physician**													**2**
**Specialist physician**													**1**
**Pharmacist**													**1**
**Pharmacist (other pharmacy)**													**1**
**Home and Community Care Nurse**													**3**
**Community Health Centre Nurse**													**3**
**Hospital ward doctor**													**1**
**Hospital ward nurse**													**1**
**Home worker**													**3**
**Home worker supervisor**													**2**
**Social worker**													**1**
													**28**

**Medication Communication Activity - Communicate MONITOR: Roles and Pathways. Represents 200 pathways between 47 roles.** Light blue boxes represent one connection (e.g., Patient --> Medical Office Assistant); whereas, dark blue boxes represent two connections within roles (e.g., communication between two (or more) Medical Office Assistants within the same clinic).	Patient	Family physician	Specialist physician	Walk-in clinic doctor	Previous provider	Doctor on call	Pharmacist	Pharmacy technician	Pharmacist (other pharmacy)	Pharmacy technician (other pharmacy)	Medical Office Assistant	Specialist Medical Office Assistant	Family member	Friends	Partner	Home and Community Care Nurse	Community Health Centre Nurse	Outreach nurse	Corrections nurse	Emergency Room doctor/Physician on duty	Hospital ward doctor	Hospital ward nurse	Hospital specialist	Hospital Home and Community Care Liaison	Community care discharge planner	Hospital records	Hospital pharmacist	Laboratory	Home laboratory services	Acute residential care	Longterm care	Home worker	Home worker supervisor	Community Health Centre Support Group	Support Group	Social worker	Case manager	Assertive Community Treatment (ACT) Caseworker	Counselor	Mental Health worker	Housing staff	Payor	St. Paul's Hospital	College of Physicians and Surgeons	College of Pharmacists	Pharmaceutical Manufacturers	Researcher	
**Patient**																																																30
**Family physician**																																																22
**Specialist physician**																																																11
**Walk-in clinic doctor**																																																3
**Previous provider**																																																1
**Doctor on call**																																																1
**Pharmacist**																																																20
**Pharmacy technician**																																																4
**Pharmacist (other pharmacy)**																																																3
**Pharmacy technician (other pharmacy)**																																																1
**Medical Office Assistant**																																																3
**Specialist Medical Office Assistant**																																																1
**Family member**																																																6
**Friends**																																																1
**Partner**																																																1
**Home and Community Care Nurse**																																																13
**Community Health Centre Nurse**																																																12
**Outreach nurse**																																																2
**Corrections nurse**																																																1
**Emergency Room doctor/Physician on duty**																																																6
**Hospital ward doctor**																																																8
**Hospital ward nurse**																																																7
**Hospital specialist**																																																5
**Hospital Home and Community Care Liaison**																																																1
**Community care discharge planner**																																																1
**Hospital records**																																																4
**Hospital pharmacist**																																																1
**Laboratory**																																																3
**Home laboratory services**																																																1
**Acute residential care**																																																1
**Longterm care**																																																1
**Home worker**																																																3
**Home worker supervisor**																																																1
**Community Health Centre Support Group**																																																2
**Support Group**																																																1
**Social worker**																																																2
**Case manager**																																																2
**Assertive Community Treatment (ACT) Caseworker**																																																2
**Counselor**																																																1
**Mental health worker**																																																3
**Housing staff**																																																1
**Payor**																																																1
**St. Paul's Hospital**																																																2
**College of Physicians and Surgeons**																																																1
**College of Pharmacists**																																																1
**Pharmaceutical Manufacturers**																																																1
**Researcher**																																																1
																																																200

**Medication Communication Activity - COORDINATE Communication: Roles and Pathways. Represents 36 pathways between 15 roles.** Light blue boxes represent one connection (e.g., Patient --> Medical Office Assistant); whereas, dark blue boxes represent two connections within roles (e.g., communication between two (or more) Medical Office Assistants within the same clinic).	**Patient**	**Family physician**	**Specialist physician**	**Walk-in clinic doctor**	**Previous provider**	**Pharmacist**	**Medical Office Assistant**	**Walk-in clinic Medical Office Assistant**	**Specialist Medical Office Assistant**	**Previous Provider Medical Office Assistant**	**Family member**	**Home and Community Care Nurse**	**Community Health Centre Nurse**	**Home laboratory services**	**Payor**	
**Patient**																**3**
**Family physician**																**1**
**Specialist physician**																**1**
**Walk-in clinic doctor**																**1**
**Previous provider**																**1**
**Pharmacist**																**1**
**Medical Office Assistant**																**13**
**Walk-in clinic Medical Office Assistant**																**3**
**Specialist Medical Office Assistant**																**4**
**Previous provider Medical Office Assistant**																**2**
**Family member**																**2**
**Home and Community Care Nurse**																**1**
**Community Health Centre Nurse**																**1**
**Home laboratory services**																**1**
**Payor**																**1**
																**36**
